# IEIVariantFilter: a bioinformatics tool to speed up genetic diagnosis of inborn errors of immunity patients

**DOI:** 10.1093/nargab/lqaf069

**Published:** 2025-05-28

**Authors:** Juan Pereda, Rafael Espinosa, Blanca García-Solís, Teresa Guerra-Galán, Ana Van-Den-Rym, Meltem Ece Kars, Rocío Mena, Victor Galán, Ana de Andrés-Martín, Carlos Rodríguez-Gallego, Alberto López-Lera, Fernando Corvillo, Antonio Pérez-Martínez, Eduardo López-Collazo, Silvia Sánchez-Ramón, Rubén Martínez-Barricarte, Lluis Quintana-Murci, José Miguel Lorenzo-Salazar, Yuval Itan, Carlos Flores, Rebeca Pérez-de-Diego

**Affiliations:** TECH ID Solutions, Madrid 28049, Spain; TECH ID Solutions, Madrid 28049, Spain; Laboratory of Immunogenetics of Human Diseases, IdiPAZ Institute for Health Research, La Paz University Hospital, Madrid 28046, Spain; Innate Immunity Group, IdiPAZ Institute for Health Research, La Paz University Hospital, Madrid 28046, Spain; Interdepartmental Group of Immunodeficiencies, Madrid, Spain; Clinical Immunology Department, San Carlos Clinical Hospital, Madrid 28040, Spain; Laboratory of Immunogenetics of Human Diseases, IdiPAZ Institute for Health Research, La Paz University Hospital, Madrid 28046, Spain; Innate Immunity Group, IdiPAZ Institute for Health Research, La Paz University Hospital, Madrid 28046, Spain; Interdepartmental Group of Immunodeficiencies, Madrid, Spain; The Charles Bronfman Institute for Personalized Medicine and Department of Genetics and Genomic Sciences, Icahn School of Medicine at Mount Sinai, New York, NY 10029, United States; Institute of Medical & Molecular Genetics (INGEMM), Hospital Universitario La Paz, Universidad Autónoma de Madrid, IdiPAZ, Madrid 28046, Spain; Translational Research in Paediatric Oncology, Haematopoietic Transplantation and Cell Therapy, IdiPAZ Institute for Health Research, La Paz University Hospital, Madrid 28046, Spain; Department of Immunology, Ramón y Cajal Hospital, Madrid 28034, Spain; Department of Immunology, University Hospital of Gran Canaria Dr. Negrin, Las Palmas de Gran Canaria 35010, Spain; CIBER de Enfermedades Respiratorias (CIBERES), Instituto de Salud Carlos III, Madrid, Spain; Department of Medical and Surgical Sciences, School of Medicine, University of Las Palmas de Gran Canaria, Las Palmas de Gran Canaria 35016, Spain; Department of Clinical Sciences, University Fernando Pessoa Canarias, Las Palmas de Gran Canaria 35450, Spain; IdiPAZ Institute for Health Research, La Paz University Hospital, CIBERER U-754, Madrid 28046, Spain; IdiPAZ Institute for Health Research, La Paz University Hospital, CIBERER U-754, Madrid 28046, Spain; Translational Research in Paediatric Oncology, Haematopoietic Transplantation and Cell Therapy, IdiPAZ Institute for Health Research, La Paz University Hospital, Madrid 28046, Spain; Innate Immunity Group, IdiPAZ Institute for Health Research, La Paz University Hospital, Madrid 28046, Spain; Interdepartmental Group of Immunodeficiencies, Madrid, Spain; Clinical Immunology Department, San Carlos Clinical Hospital, Madrid 28040, Spain; Division of Genetic Medicine, Department of Medicine, Vanderbilt Genetics Institute, Vanderbilt University Medical Center, Nashville, TN 37232, United States; Division of Molecular Pathogenesis, Department of Pathology, Microbiology, and Immunology, Vanderbilt Center for Immunobiology, Vanderbilt Institute for Infection, Immunology, and Inflammation, Vanderbilt University Medical Center, Nashville, TN 37232, United States; Unit of Human Evolutionary Genetics, Institut Pasteur and CNRS URA3012, Paris 75015, France; CIBER de Enfermedades Respiratorias (CIBERES), Instituto de Salud Carlos III, Madrid, Spain; Genomics Division, Instituto Tecnológico y de Energías Renovables (ITER), Santa Cruz de Tenerife 38600, Spain; The Charles Bronfman Institute for Personalized Medicine and Department of Genetics and Genomic Sciences, Icahn School of Medicine at Mount Sinai, New York, NY 10029, United States; CIBER de Enfermedades Respiratorias (CIBERES), Instituto de Salud Carlos III, Madrid, Spain; Department of Clinical Sciences, University Fernando Pessoa Canarias, Las Palmas de Gran Canaria 35450, Spain; Genomics Division, Instituto Tecnológico y de Energías Renovables (ITER), Santa Cruz de Tenerife 38600, Spain; Research Unit, Hospital Universitario Ntra. Sra. de Candelaria, Instituto de Investigación Sanitaria de Canarias (IISC), Santa Cruz de Tenerife 38010, Spain; Laboratory of Immunogenetics of Human Diseases, IdiPAZ Institute for Health Research, La Paz University Hospital, Madrid 28046, Spain; Innate Immunity Group, IdiPAZ Institute for Health Research, La Paz University Hospital, Madrid 28046, Spain; Interdepartmental Group of Immunodeficiencies, Madrid, Spain; CIBER de Enfermedades Respiratorias (CIBERES), Instituto de Salud Carlos III, Madrid, Spain

## Abstract

Severe infectious diseases remain the leading cause of death in children and young adults worldwide. Monogenic inborn errors of immunity (IEIs) are traditionally defined as a heterogeneous group of rare inborn genetic diseases affecting the functioning of the immune system. Greater awareness has led to the clinical definition of 485 monogenic IEIs and whole exome sequencing (WES) is becoming increasingly relevant for IEI genetic diagnosis. The current protocol for IEI genetic studies includes manual filtering of the list of genes obtained as a WES read-out providing a short list of candidate genes. This procedure is time-consuming and can produce mistakes due to human error in manual filtering. IEIVariantFilter is a new web-based bioinformatics tool to speed up and refine the genetic diagnosis of IEI patients oriented for users in the biomedical field without needing bioinformatics expertise. IEIVariantFilter prioritizes genetic variants based on ranges of zygosity, the quality of reads, the predicted variant effect, and genes related to immunity, considering a consanguineous hypothesis whenever necessary. IEIVariantFilter facilitates gene and variant list prioritization, speeding up the identification of candidate disease-causing variants for validation by experimental studies. The software improves the genetic diagnosis of patients, thereby facilitating precision medicine and fast and proper treatment.

## Introduction

Inborn errors of immunity (IEIs) are a heterogeneous group of diseases caused by quantitative and/or functional changes in the different mechanisms involved in both the innate and the adaptive immune responses [1]. They are classified as primary immunodeficiencies (PIDs), when their origin is genetic, and secondary immunodeficiencies (SIDs), when their origin is acquired. Both types are associated with or cause a predisposition towards clinical complications, such as severe or recurrent infections, autoimmune disorders, immune dysregulation with lymphoproliferation, inflammatory disorders, lymphomas, and other types of cancer, many of which are diagnosed and treated by rheumatologists, haematologists, and oncologists [1–5]. IEIs were traditionally defined as rare inborn genetic diseases affecting the functioning of the immune system. However, it has become apparent in recent years that IEIs are much more common than initially thought, and a high percentage of patients with severe infections since childhood have an IEI [2, 3, 6]. Greater awareness and improved collaboration in recent years have led to the clinical definition of 485 monogenic IEIs [1]. IEI studies have greatly improved our understanding of human immunology, providing a framework for studies of physiologically relevant genes in the human context [4, 7]. In addition, whole exome sequencing (WES), i.e. the targeted sequencing of the protein-coding portion of the human genome, has proven to be a powerful and efficient method for identifying disease-causing variants underlying Mendelian disorders [8]. It has been estimated that the protein-coding regions of the human genome account for ∼85% of all described disease-causing variants [9]. The use of WES is becoming increasingly relevant for IEI genetic diagnosis as our understanding of the functional consequences of sequence variation improves [9]. Researchers who typically confront the WES data analyses of IEI patients come from the biological, chemical, medical, or pharmaceutical fields, as it is their knowledge of the biology of the immune system that yields new candidate genes to find new genetic defects. However, the use of some software or the design of scripts for filtering requires bioinformatics skills that prevent these researchers from using bioinformatics tools, which increases the risk of errors in the first steps of WES filtering.

Despite recent advances, the genetic basis of a significant proportion of IEIs remains undefined. The continuous improvement of technologies for the genetic diagnosis of diseases is unprecedented and the impact of this exponential improvement is further amplified when these technologies coalesce into open platforms and ecosystems. For instance, new categories of technologies in molecular biology and materials science are being combined, leading to advances and radical changes in approaches in an ever-increasing number of industry functions and fields. Several algorithms have been developed to help in WES data interpretation, such as the CADD (Combined Annotation Dependent Depletion) score and the REVEL (Rare Exome Variant Ensemble Learner) that predict deleteriousness/pathogenicity of genetic variants [10, 11]. Another relevant tool to help discover a large proportion of IEI-causing genes in patients is the human gene connectome and the interactome approach, which shows the biological distance between all human genes and can be used to prioritize candidate genes by their relatedness to known disease-causing genes [12]. All these tools and algorithms, together with new high-throughput sequencing techniques, represent a powerful strategy for the identification of new genetic aetiologies of IEIs. However, even today, filtering such data is mainly performed manually, increasing the risk of human errors and slowing down the analysis. Yet in line with one of the objectives of the International Rare Diseases Research Consortium (IRDiRC) [13], IEIVariantFilter is a tool with a pipeline tailored specifically for IEI, since the genetic causes of different disease groups are extremely heterogeneous. IEIVariantFilter provides easy-to-use automatic variant filtering, offering the user without needing bioinformatics expertise a shortened list of candidate genes for further detailed study by the researcher, thus achieving faster filtering, allowing a genetic diagnosis and rapid clinical decisions for IEI patients.

## Materials and methods

### Study approval

The experimental protocol was approved by the Ethics Committee of La Paz University Hospital (Madrid, Spain) and written informed consent was obtained from the patient’s family for participation in this study.

### Platform architecture

We developed the IEIVariantFilter, a tool based on features of genetic variants obtained as a read-out of WES data from patients and controls that allows filtering of these variants. Due to the limitations of our server, the tool can only be used for WES files and not whole genome sequencing (WGS) files, which are much larger. The tool accepts a tabular-format input file containing annotated variants through a web-based graphic user interface with a number of annotation fields (see below). The user is asked to select the reference genome assembly used for the annotation (GRCh37.p13 or GRCh38.p14 are available at the moment). Only WES files from the same human genome assembly can be used for filtering.

The software uses samples as controls to filter ‘benign’ features; these samples can be healthy controls or patients with different pathology from the patient analysed (pseudo-controls).

WES result files must be uploaded to the database following the instructions in the User Guide (see supplementary material and website). It is installed on a server providing security password-controlled access for users after requesting permission.

These data will not be shared between users following data protection rules and informed consent.

### WES file filtering tool

The filtering tool was developed in PHP for the front part, in server-side scripting language for the frontend (Apache server), and in Java for communications and the backend (Tomcat server) (Supplementary Fig. S1).

The user manually inputs a gene to compare or a tabular format Excel-like files with a list of genes to compare through the graphical interface provided by the tool. Subsequently, the platform searches within the database for the correlation of any gene that matches those previously entered. Due to the size of the search, the Spring Batch framework is used, which allows processing the information in batches (Supplementary methods and Supplementary Fig. S2). Before conducting the search and applying filters, the user must select the filters to apply, which include the patient or patients, the controls, and the consanguineous parents (if applicable).

The software includes variant filtering based on (i) filtering variants by range of zygosity (heterozygous/homozygous), quality of reads, genes included in the blacklist (genes not related to immunology), 3′ and 5′ UTRs, and synonymous variants; (ii) highlighting compound heterozygous variants and genes in the white list, a list of genes involved or related to immunology (7393 genes, Supplementary Table S1) [14]; and (iii) considering recessive hypothesis homozygous filtering for the patient and heterozygous filtering for parents.

To refine the search results, the following approximations are leveraged:

- *Indexing*: We have created indices on columns that are frequently used in the WHERE, JOIN, or ORDER BY clauses of queries. These allow the database engine to efficiently find the data without need to scan the entire table.
- *Query optimization*: We have reviewed and optimized SQL queries to ensure that they are as efficient as possible. This includes selecting only the necessary columns, avoiding unnecessary subqueries, and using appropriate joins.
- *Result caching*: We have implemented a caching solution to store the results of frequently executed queries. This helps to significantly reduce the response time for these queries by avoiding the need to access the database each time.

## Results

### Access to the software website, uploading WES files, and filtering steps

The software is accessible at the following URL: http://185.49.184.146:8080/exome-web/login?redirect=%2Fapp%2Fdashboard. As the web server is paid for our lab projects, its capacity is limited, and user access must be requested. Upon register via the webpage, a user will be created, and credentials will be provided by email. Users can then upload the WES files of their samples. Each user will be allowed to analyse their own uploaded data. The data entered by each user are private and can only be accessed by the user themselves, ensuring that the results remain completely confidential. Upon request to the correspondence author, users can be connected to each other confidentially so that they can share data.

**Figure 1.**
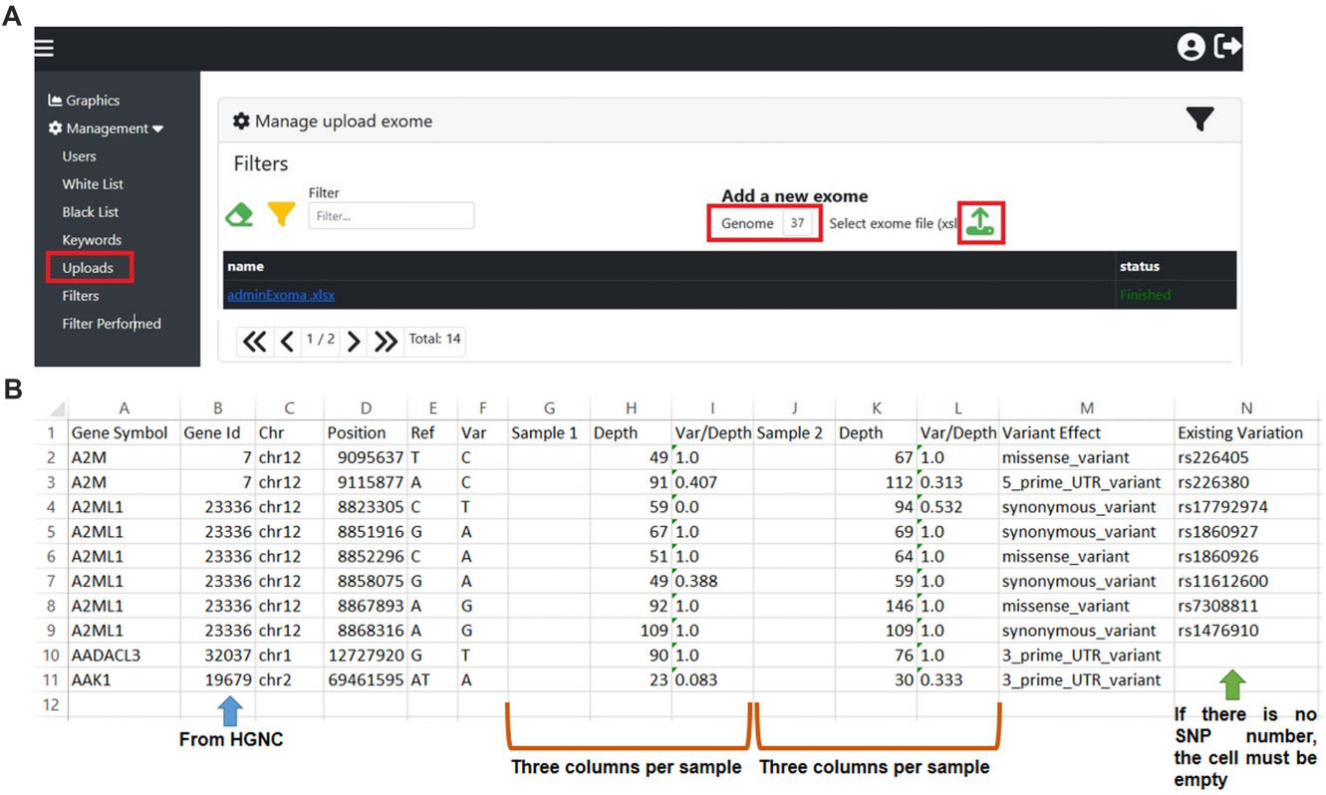
Uploading WES files. (**A**) Software interface highlighting the uploading of WES files in red. (**B**) Format for .xlsx files to be uploaded. The columns should contain the following data: *Gene Symbol*; *Gene Id* (from HGNC); *Chr* (chromosome); *Position* (position number in the genome); *Ref* (reference allele); and *Var* (variant allele). Three more columns contain the data from each sample: *Sample 1* (patient identification, this column is empty); *Depth* (number of total reads of sample 1 for the variant indicated in each file); and *Var/Depth* (allele balance of sample 1 for the variant indicated in each file). Another column contains the *Variant Effect* (if the variant effect is 5′ UTR, 3′ UTR, or a synonymous variant, it should be indicated exactly in these formats: 5_prime_UTR_variant, 3_prime_UTR_variant, or synonymous_variant) and *Existing Variation* (if there is no SNP number, the box must be left empty).

**Figure 2.**
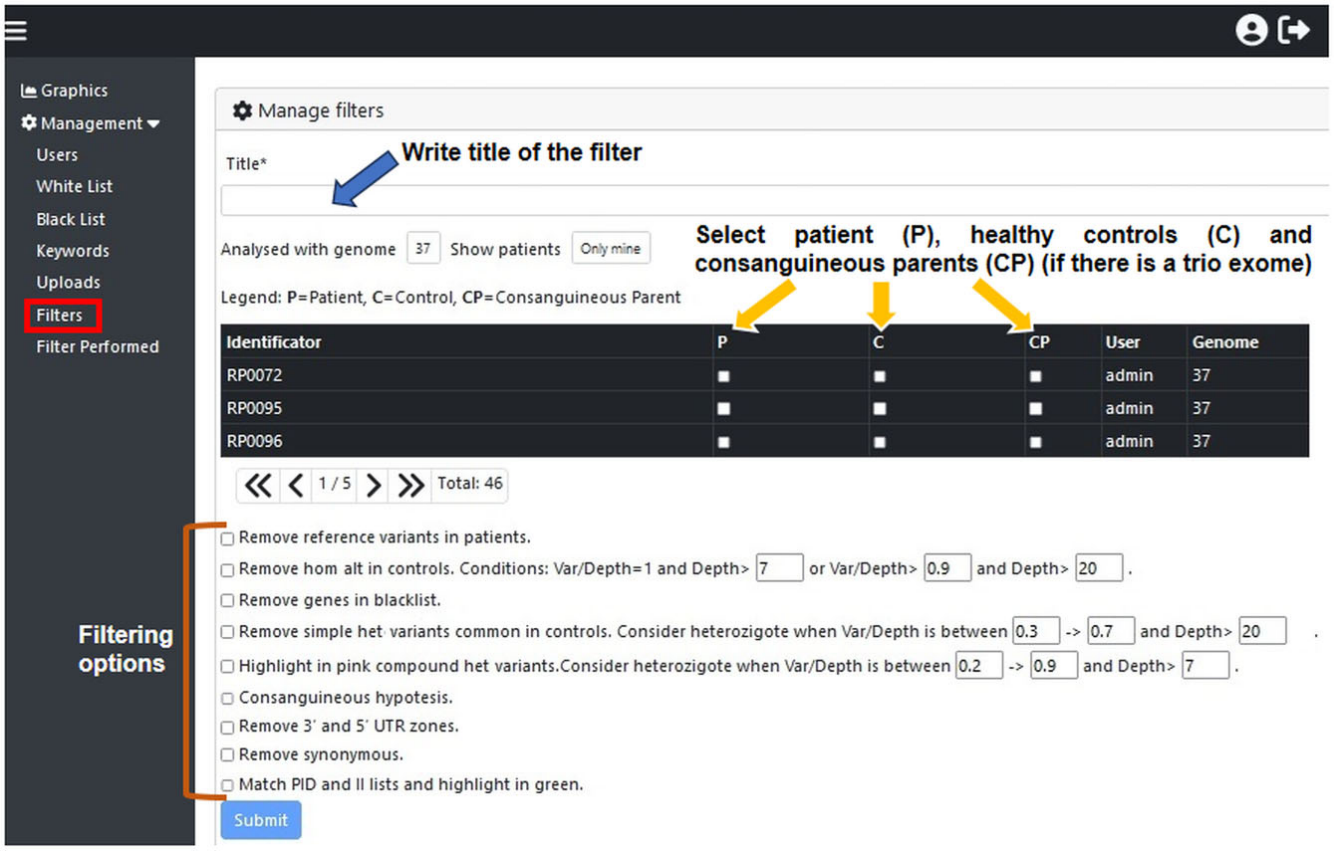
Filtering of WES samples. WES samples are filtered in the *Filters* option. After naming the filter, patients (P), controls (C), and consanguineous parents (CP) (the latter in the case of a Trio WES with hypothesis of consanguinity) are selected. Different checkboxes (yellow arrows in the figure) allow WES files to be filtered (see Results: ‘Steps for filtering’ for a description of each).

Tabular format (Excel files) input files can be filtered to exclude common variants and intronic variants beforehand for faster uploading. A User Guide provides detailed instructions for uploading WES files and the recommended steps for filtering (see supplementary material or website; Figs 1 and 2).

### Downloading results after filtering

**Figure 3.**
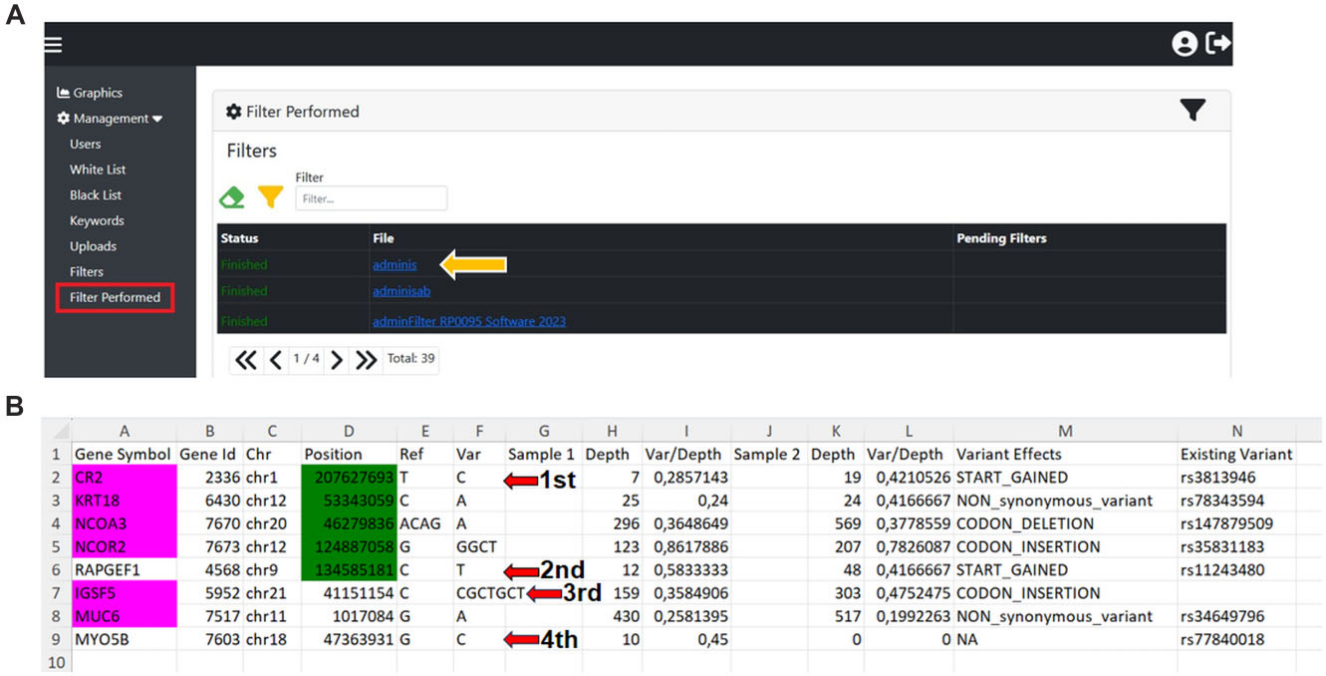
Filter performed. (**A**) Software interface for the *Filter Performed* section. Files are downloaded in Excel-like format (yellow arrow). (**B**) The Excel spreadsheet obtained after filtering, organized in the subsequent columns: *Gene Symbol*; *Gene Id*; *Chr*; *Position*; *Ref*;and *Var*. The next three columns show the data for each subject: *Sample* (patient identification); *Depth* (number of total sample reads); *Var/Depth* (allele balance); *Variant Effect*; and *Existing Variation*. Green boxes indicate genes in the white list and pink boxes indicate heterozygous compound variants.

After defining and submitting the required filters, the tool provides a filtered list of variants that is downloadable from the *Filter Performed* section (Fig. 3). The User Guide (see the supplementary material or website) describes how the list of filtered variants is returned to the user.

### Case study on a patient with IEI

**Figure 4.**
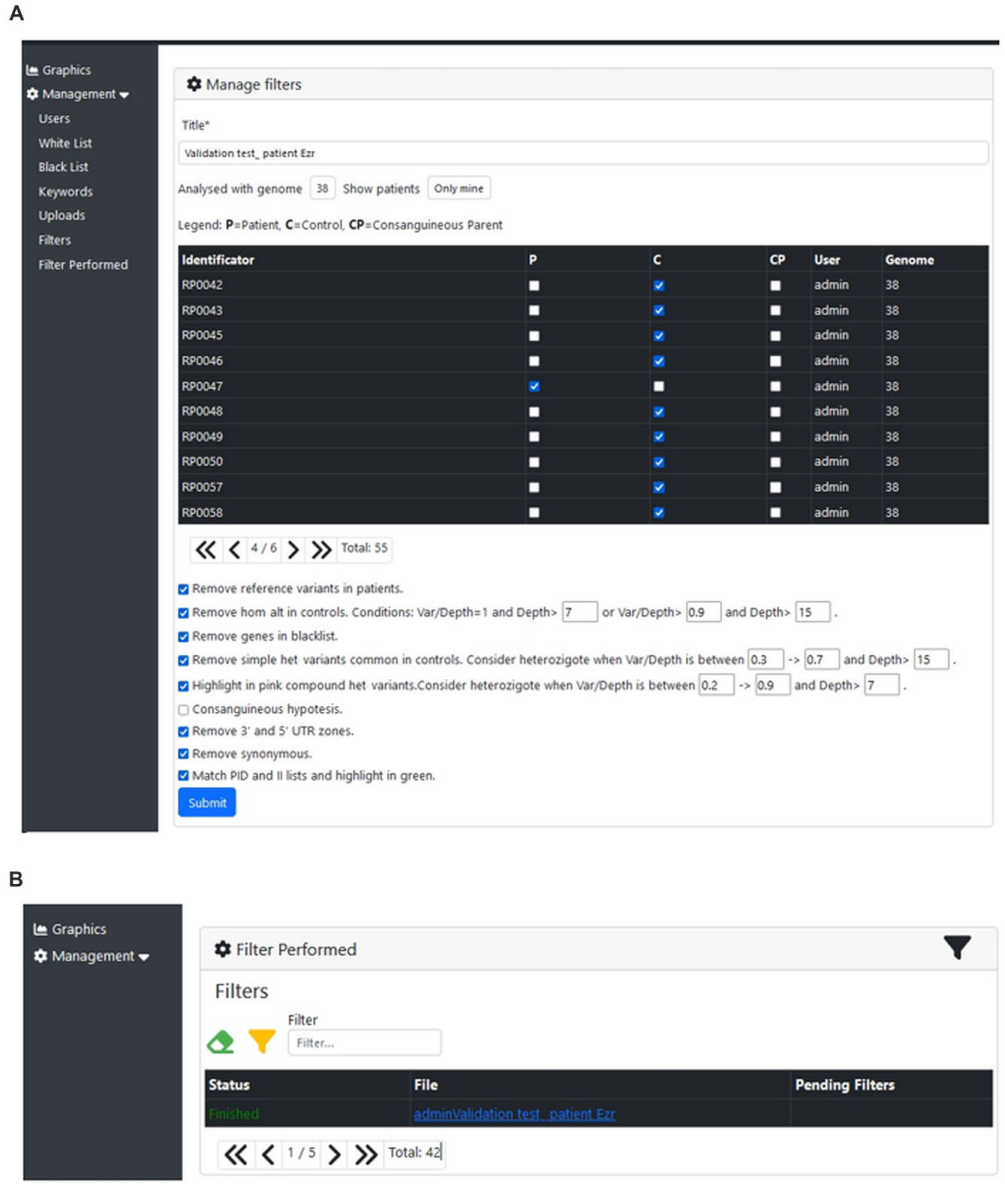
Case study on a patient with IEI. (**A**) Software user interface for filtering data from a patient reported as a carrier of the *EZR* deficiency [15]. (**B**) Result of data filtering for the *EZR* carrier patient in the validation test.

We tested the software with WES data from a patient with a known homozygous disease-causing variant in *EZR* [15]. The patient has consanguineous parents, but WES data were lacking from the parents. In *Manage Filters*, we selected the patient (ID: RP0047) and available data from several samples to serve as controls (Fig. 4A). The following options were selected for filtering:

- *Remove reference variants in patients*: This removed variants that were homozygous reference in the patient analysed.
- *Remove hom alt in controls:* This removed homozygous variants in controls, as the premise establishes that a homozygous variant in unaffected cannot be responsible for the disease in the patient.
- *Remove genes in the blacklist*: This checkbox removed all variants of genes included in the blacklist.
- *Remove simple het variants common in controls*: This removed variants in heterozygosis that were present in patients and in controls; the premise is that a simple heterozygous variant in a patient that appears in unaffected is not responsible for the disease.
- *Highlight in pink compound het variants*: This highlights in pink colour the variants that are compound heterozygous at the range of Var/Depth ratio and depth established by the user.
- *Remove 3′ and 5′ UTR zones* and *remove synonymous*.
- *Match PID and II list* and *highlight in green*: This checks which variants are in genes that match a white list of genes related to PID, innate immunity, and infectious diseases.

After submitting the filtering, the result is downloaded from *Filter Performed* (Fig. 4B). For this patient, a total of 1770 variants were obtained (Supplementary Table S2). From these, 592 variants were highlighted in green, as they were genes related to immunology. Since the patient is consanguineous, we selected the homozygous variants highlighted in the white list and then eight variants were obtained (Table 1A). Further filtering of these eight variants in terms of quality of reads (we removed variants with <10 reads) left us with four variants, one in each of the following genes: *MAML2*, *LILRB3*, *EZR*, and *MAP3K4* (Table 1B). The clinical features of the patient fitted the *EZR* variant better, making it the best candidate gene for downstream analyses.

**Table 1.** Case study on a patient with IEI

A										
Gene symbol	Gene Id	Chr	Position	Ref	Var	RP0047	Depth	Var/ Depth	Variant effects	Existing variant
*HLA-B*	4932	chr6	31356431	G	A	P_Homo_var	2	1	missense_variant	Null
*PDE4DIP*	15580	chr1	148870282	G	T	P_Homo_var	2	1	missense_variant	rs12118314
*PDE4DIP*	15580	chr1	148870283	T	G	P_Homo_var	2	1	missense_variant	rs12119750
*MAML2*	16259	chr11	96093510	C	T	P_Homo_var	36	1	missense_variant	rs61749254
*LILRB3*	6607	chr19	54222457	T	C	P_Homo_var	14	1	missense_variant	rs200199363
*EZR*	12691	chr6	158785391	C	T	P_Homo_var	29	1	missense_variant	rs528409234
*MAP3K4*	6856	chr6	161070652	G	T	P_Homo_var	21	1	missense_variant	rs34018542
*SRP9*	11304	chr1	225786860	A	T	P_Homo_var	1	1	splice_acceptor_variant	Null
**B**										
*MAML2*	16259	chr11	96093510	C	T	P_Homo_var	36	1	missense_variant	rs61749254
*LILRB3*	6607	chr19	54222457	T	C	P_Homo_var	14	1	missense_variant	rs200199363
*EZR*	12691	chr6	158785391	C	T	P_Homo_var	29	1	missense_variant	rs528409234
*MAP3K4*	6856	chr6	161070652	G	T	P_Homo_var	21	1	missense_variant	rs34018542

(**A**) Homozygous variants present in the white list of immune genes for the patient analysed in the validation test. (**B**) Homozygous variants present in the white list of immune genes, removing those with a low number of reads. Ref: reference nucleotide and Var: variant nucleotide.

## Discussion

IEIs are a heterogeneous group of different (>400) immunity disorders that impair the functions of the human immune system, causing greater susceptibility to infection, inflammation, autoimmunity, allergy, and malignancy. These diseases pose a challenge for diagnosis and treatment due to overlapping symptoms and similarities between diseases. IEIs are thought to affect at least 10 million people worldwide [16]. WES/WGS combined with the existing knowledge can provide a genetic aetiology around 50% of IEI patients assessed [17]. Therefore, IEIs should no longer be considered as rare diseases due to the number of undiagnosed patients. The International Union of Immunological Societies Inborn Errors of Immunity Committee only reports single-gene inborn errors [1]. However, IEI experts around the world suspect that the high percentage of undiagnosed patients is due to complex genetic scenarios such as oligo- or polygeny. If IEIs are left un/misdiagnosed, the immune system remains compromised, leading to chronic illness, disability, reduced working capacity, decreased quality of life for patients and families, permanent organ damage, or even death. Despite the clear relevance of these disorders, there is still an urgent need for a better understanding of their causes and early detection, to design better therapies, improve prognosis, and provide accurate genetic counselling to the family. The current procedure for IEI studies includes filtering the list of genes obtained as a WES/WGS read-out, resulting in a short list of candidate genes that are then experimentally validated. This procedure is slow and can lead to human errors associated with manual filtering.

**Figure 5.**
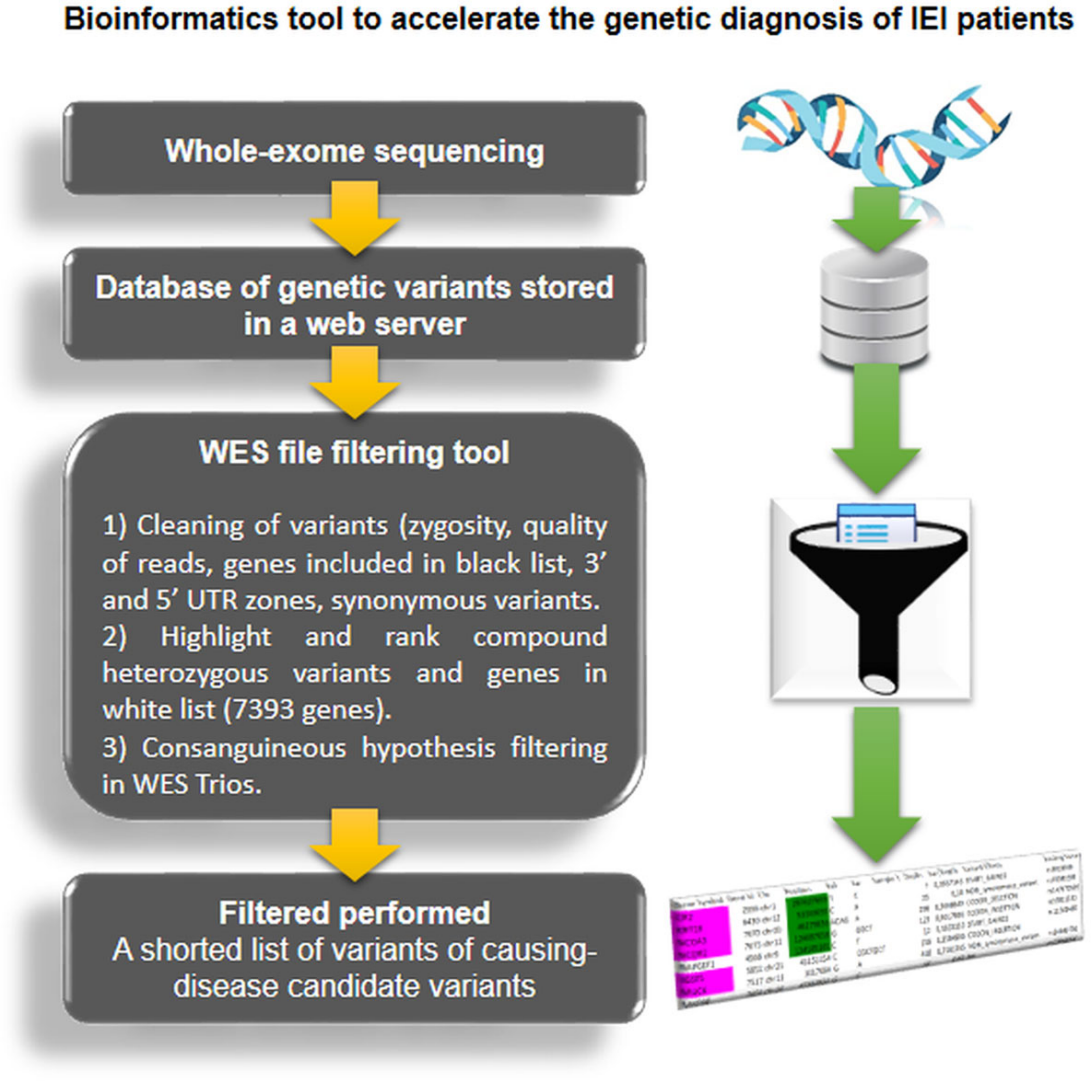
Flowchart of IEIVariantFilter to speed up genetic diagnosis of IEI patients.

The researchers who typically analyse the IEI patients’ WES data come from the biological, chemical, medical, or pharmaceutical fields, and their knowledge of the human immune system biology is leveraged to filter the WES-provided list of variants to find new genetic defects. In many cases, the researchers have difficulties to deal with complex bioinformatics tools or to develop scripts for WES filtering, and they have to make all filtering process by hand, increasing the risk of errors. IEIVariantFilter is developed to facilitate the analysis of these datasets by these researchers. The bioinformatics tool speeds up and refines the genetic diagnosis of IEI patients. With this easy-to-use tool, the user can filter and prioritize genetic variants based on ranges of zygosity, quality of reads, and predicted variant effect. The software also highlights compound heterozygous variants and genes related to immunology (contained in the white list) and excludes those not related to immunology. It can also consider recessive hypotheses. This new web-based tool significantly reduces the list of variants for final manual filtering, speeding up the selection of disease-causing candidate variants for experimental validation (Fig. 5). In our experience, we reduced the list of candidate variants by 10-fold compared to the standard manual filtering. In addition, the filtering tool can be coupled to future tools that allow the study of more complex scenarios, such as polygenic hypotheses or incomplete penetrance, by linking the tool to the connectome [12].

*Software limitations*: The software is prepared for IEI study, not for general use. In addition, the web server has size limitations, so it is necessary to include only the annotation files indicated in the User Guide. Also, the software does not include filtering based on allele frequency, so it is recommended to include only the allele frequency variants that the user wants to analyse.

## Supplementary Material

lqaf069_Supplemental_Files

## Data Availability

IEIVariantFilter is available at http://185.49.184.146:8080/exome-web/login?redirect=%2Fapp%2Fdashboard.
