# Supplementary material for: IEIVariantFilter: a bioinformatics tool to speed up genetic diagnosis of inborn errors of immunity patients
Source: NAR Genom Bioinform. 2025 May 28;7(2):lqaf069. doi: 10.1093/nargab/lqaf069 (PMC12117399; doi:10.1093/nargab/lqaf069)

**Supplementary Online Material Contents**

**Methods: Spring Batch**

**Figure S1**

**Figure S2**

**Tables Legend S1 and S2**

**USER GUIDE**

**Methods: Spring Batch**

We devised *IEIVariantFilter* to search for one or several genes in a database larger than 96 GB (information preloaded through batch process). To do this, we examined the applicability and effectiveness of the Spring Batch framework (<https://spring.io/projects/spring-batch>), an open-source framework which is oriented towards processing large volumes of data. Spring Batch is recognised for its ability to handle batch-based operations, which facilitates the management of data segments independently, a key functionality for working with large files (Fig. S1).

In terms of scalability, Spring Batch offers a model that allows the distribution of processing across multiple nodes. This feature is essential for parallelising search and processing operations, resulting in a significant reduction in the time required for such operations.

The Spring Batch restart and recovery capability is also of great relevance. In long processes involving large volumes of data, the ability to resume processing from the last known point in the event of failure is crucial to ensuring the integrity and continuity of the data.

Additionally, support for transactions ensures data consistency during processing. This property is vital when handling large amounts of information, where any error could result in significant inconsistencies.

Spring Batch also incorporates strategies to optimise performance, such as multithreading and pagination, which are fundamental for efficient resource management and reducing processing time in large files.

Finally, the Spring Batch integration and extensibility capabilities allow the framework to be adapted to various needs and specific project requirements, offering considerable flexibility to implement customised functionalities.

**FIGURES**

**Figure S1. Architecture of the filtering tool in *IEIVariantFilter*.**

**
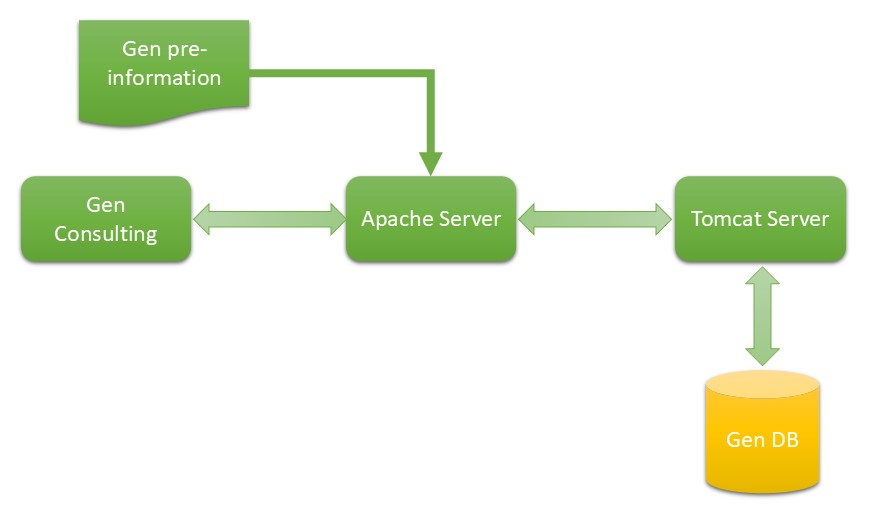
**

**Figure S1.** **Architecture of the filtering tool in *IEIVariantFilter*.**

The front part was developed in PHP, the frontend was developed in an Apache Server, and the communications and the backend components were developed using Java (Tomcat server).

**Figure S2. Spring Batch Architecture.**

**
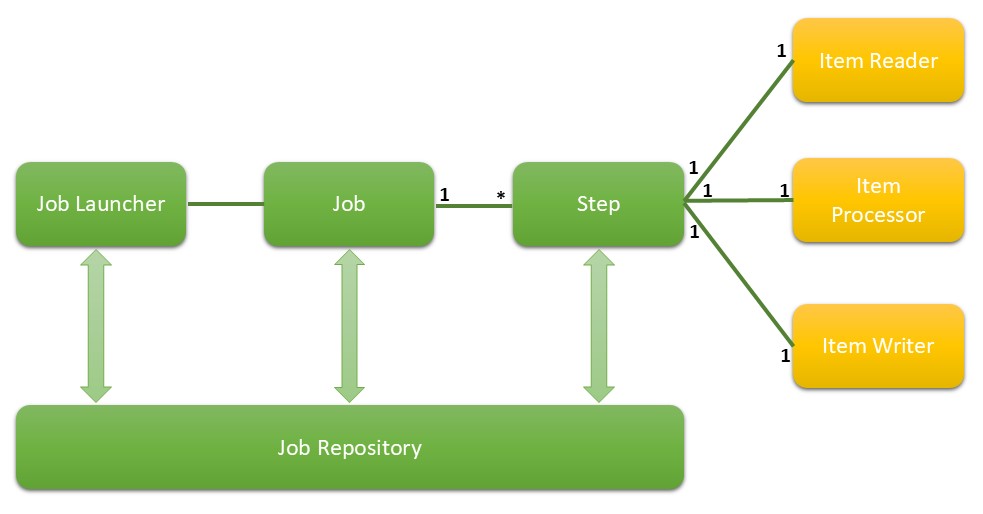
**

**Figure S2. Spring Batch Architecture.**

Spring Batch Architecture used for *IEIVariantFilter* (https://spring.io/projects/spring-batch). ‘1 to *’ means one job can perform several steps. ‘1 to 1’ means one job can perform a single step (read, execute or write).

**TABLE LEGEND**

**Table S1. White list.** Immunology-related genes.

**Table S2. Validation study.** Result downloaded from *Filter Performed* for the patient analysed in the validation study. A total of 1,770 variants were initially obtained before additional filtering steps.

**USER GUIDE**

**Uploading WES data files into *IEIVariantFilter***

- *IEIVariantFilter* accepts tabular format input files containing previously called and annotated variants. Only .xlsx files can be uploaded. The user must define whether WES data is in GRCh37.p13 or GRCh38.p14 reference genome coordinates. Only WES data files from the same human assembly can be used for the filtering.
- WES data files must comprise the annotation fields: *Gene Symbol*, *Gene Id* (from HGNC), *Chr*, *Position*, *Ref* (reference nucleotide), *Var* (variant nucleotide), *Patient* *Identification*, *Depth* (number of total reads), *Var/Depth* (allele balance), *Variant Effect*, and *Existing Variation* (rs number). The columns *Patient Identification*, *Depth*, and *Var/Depth* are added for each sample.


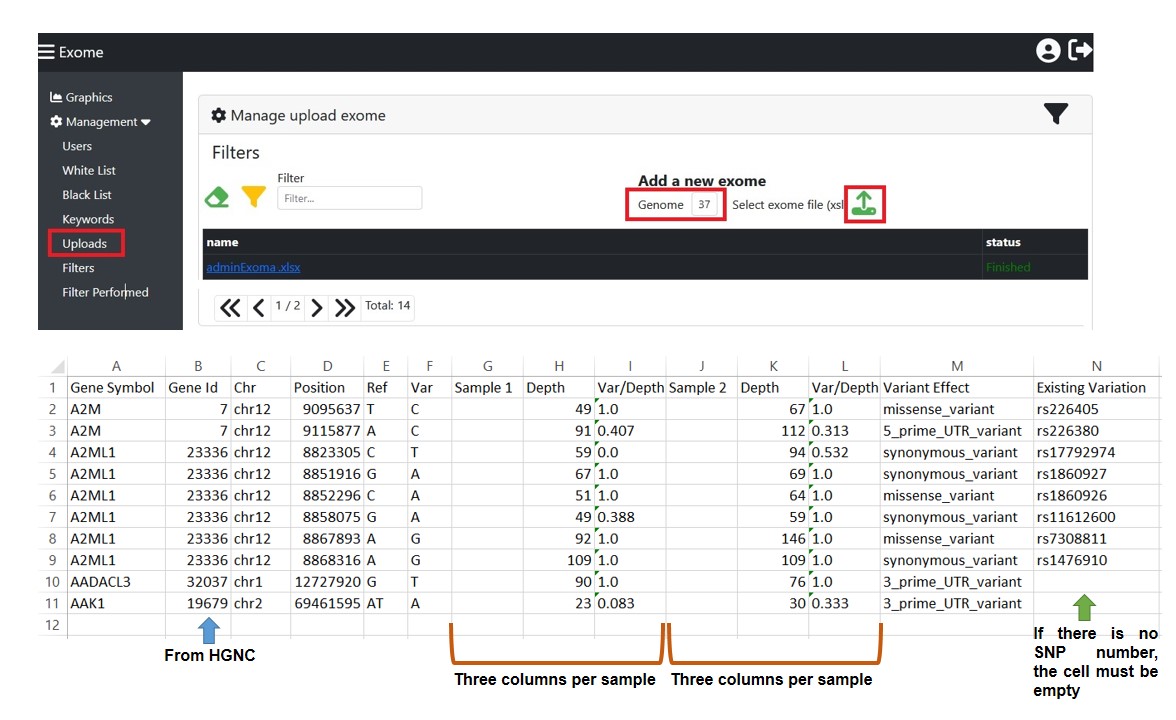


**Steps for filtering:**

- The *filters* option lets the user filter one or more WES files. The user must name the filter and select which samples are patient or patients (P), controls (C), and consanguineous parents (CP). For the datasets identified as patients (P), the user can select more than one in cases of siblings with the same pathology; controls (C) can be WES data from healthy donors or from patients with a disease different from the patient being analysed (unaffected for the disease under study in any case). **The use of these controls helps to filter and eliminate common repetitive variants in the exomes.** Finally, the consanguineous parents (CP) filter is used when there is a Trio WES (patient and parents) with consanguinity hypothesis. If CP samples are selected, the check box ‘Consanguineous hypothesis’ must also be selected. The software will filter to show only homozygous variants in P that are heterozygous variants in CP.
- Several check boxes can be used to filter the WES samples:
  - *Remove reference variants in patients*: This removes variants which are homozygous reference in the analysed patient.
  - *Remove hom alt in controls:* This removes homozygous variants in controls, as the premise establishes that a homozygous variant in unaffected individual cannot be responsible for the disease in the patient (there is a range that can be modified to establish which Var/Depth ratio is considered homozygous and how many reads (Depth) are enough for a good quantity of reads).
  - *Remove genes in the black list*: This removes all variants of genes included in the black list. This list can be added in the *black list* section and is added by the HGNC number to avoid duplicating gene names.
  - *Remove simple het variants common in controls*: This removes variants in heterozygosis that are present in patient and controls; the premise is that a simple heterozygous variant in a patient that appears in an unaffected control is not responsible for the disease. The software only removes simple heterozygous variants, checking that there is only one variant in the gene analysed. If there is more than one, this filter is not applied to avoid eliminating compound heterozygotes. Again, there is a range that can be modified to establish which Var/Depth ratio is considered heterozygous and how many reads (Depth) are enough for a good quantity of reads.
  - *Highlight compound het variants in pink*: This highlights in pink the compound heterozygous variants with the range of Var/Depth ratio and depth established by the user. For a specific gene, the software detects whether there is more than one variant with a different position, ref and var, that complies with the established range.
  - *Remove 3’ and 5’ UTR zones* and *remove synonymous*: These check boxes remove variants that are UTR or synonymous variants. It is important that the format is indicated in the column variant effect as 5_prime_UTR_variant, 3_prime_UTR_variant or synonymous_variant, so the software can recognise these variants.
  - *Match PID and II list and highlight in green*: This checks which variants are in genes matched with a white list that are related to primary immunodeficiencies (PID), innate immunity and infectious disease. The default list includes 7,393 genes and more genes can be added by the user. These genes will be highlighted in green and are ranked first in the list of filtered variants. The filtering is performed sequentially.

*
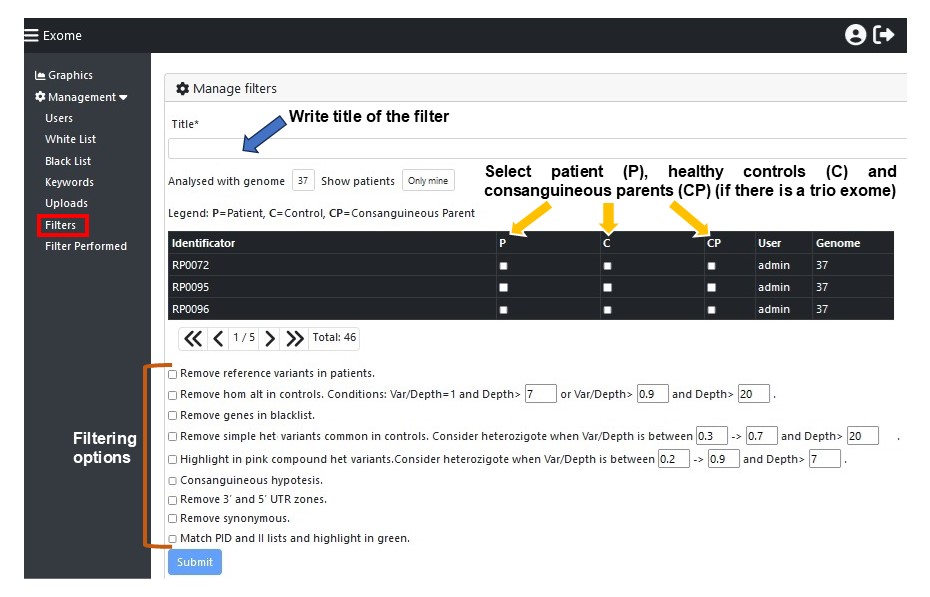
*

**Filter performed**

- After submitting the required filter, the results are downloaded in the *Filter Performed* section in Excel format. The Excel spreadsheet obtained contains the following columns: *Gene Symbol*, *Gene Id*, *Chr*, *Position*, *Ref*, *Var*, followed by three columns with the data for each sample: *Sample* (patient identification), *Depth*, *Var/Depth*, *Variant Effect* and *Existing Variation*. All samples included in the analysis are shown. Results will be ranked by assigning the first rows to those genes existing in the white list (marked in green in the first column) and highlighted as heterozygous compound variants (marked in pink in the second column). The second set of rows correspond to genes only existing in the white list (in green). Third are the genes highlighted as heterozygous compound variants (in pink). Finally, variants with no previous conditions are shown.


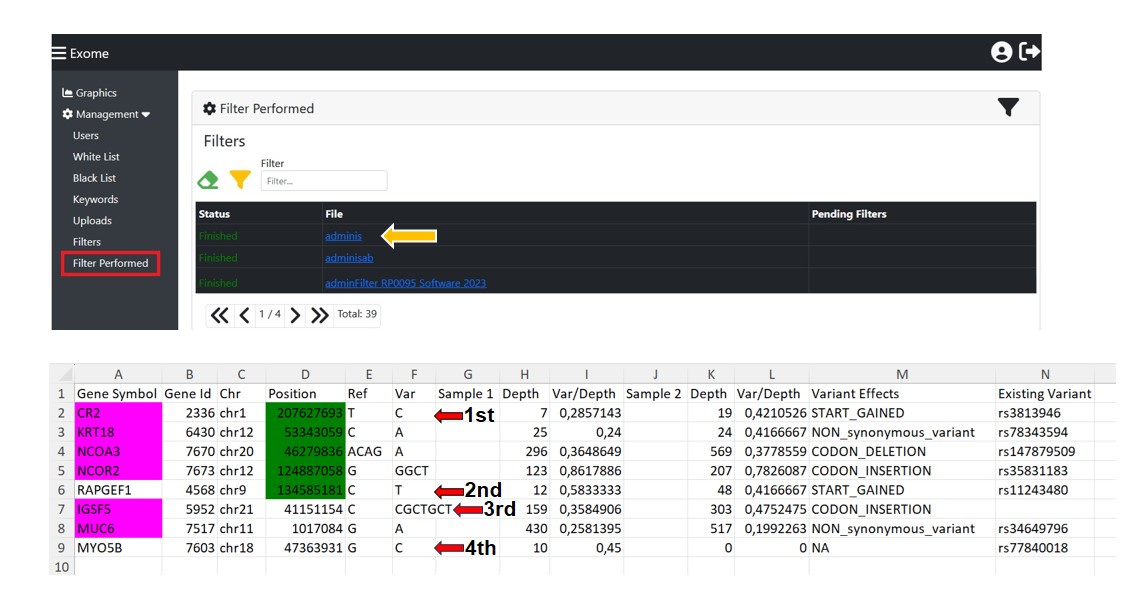

Supplement: lqaf069_Supplemental_Files [file lqaf069_supplemental_files.zip › IEIVariantFilter SOM revised.docx]
